# Utilization of Personal Protective Equipment and Associated Factors among Large-Scale Factory Workers in Debre-Berhan Town, Amhara Region, Ethiopia, 2021

**DOI:** 10.1155/2022/8439076

**Published:** 2022-02-08

**Authors:** Muluken Tessema, Wondimu Sema

**Affiliations:** ^1^Department of Public Health, Institute of Medicine and Health Sciences, College of Health Science, Debre Berhan University, P.O. Box 445, Debre Berhan, Ethiopia; ^2^Department of Environmental Health, Debre Berhan Health Science College, Debre Berhan, P.O. Box 37, Ethiopia

## Abstract

**Background:**

Personal protective equipment was designed to protect workers from serious workplace injuries or illnesses resulting from contact with chemical, radiological, physical, electrical, mechanical, or other workplace hazards. Use of personal protective equipment has been identified as an important hazard control strategy in work environments where it may not be practical to adopt other strategies.

**Objective:**

To determine *personal protective equipment* utilization and its associated factors based on health belief model among large scale factory workers in Debre-Birhan, Ethiopia.

**Methods:**

An institution-based cross-sectional study was employed in Debre-Birhan Town, North Shoa Ethiopia, from April 1 to May 1, 2021. The data were collected by using an interviewer-administered structured questionnaire. A total of 412 samples were selected by systematic random sampling method. The data were entered to EpiData version 3.1 and analyzed by SPSS. All independent variables were fitted into the binary logistic regression model to evaluate the degree of association and variables with a *p* value of <0.2 that was fitted for multiple logistic regressions. Finally, variables with a *p* value of <0.05 was found to be statistically significant.

**Result:**

A total of 412 workers were study participants with 100% response rate. The mean age was 29 (±7.3) years. Most workers, 367 (89%) knew that PPE can prevent work-related injury and illness. Overall, 172 (41.7%) of the workers were considered to have good personal protective equipment utilization. Perceived susceptibility (AOR = 1.2, 95%, CI (1.076–1.38)), perceived severity (AOR = 1.1, 95%, CI (1.088–1.163)), perceived self-efficacy (AOR = 1.2, 95%, CI (1.082–1.349)), and perceived barrier (AOR = 0.87, 95%, CI (0.800–0.956)) were found to be significant predictors of good personal protective equipment utilization.

**Conclusion:**

The study revealed that good personal protective equipment utilization in large-scale factory workers. Perceived susceptibility, perceived severity, perceived barrier, and perceived self-efficacy were found to be predictors of PPE utilization. It is recommended that, during delivery of health education special emphasis should be given to severity, susceptibility, barrier, and self-efficacy of occupational disease.

## 1. Background

Personal protective equipment was designed to protect workers from serious workplace injuries or illnesses resulting from contact with chemical, radiological, physical, electrical, mechanical, or other workplace hazards. It may include items such as gloves, safety glasses and shoes, earplugs or muffs, hard hats, respirators, or coveralls, vests, and full body suits [1]. It is a significant determining factor between an accident and safety in the working environment. Evidence suggests that wearing the correct personal protection at all times is extremely important in reducing accidents and should be given high priority [2]. Globally, 34% of occupational accidents were resulting from the lack of use of PPE available at workplace at the time of the accident [3]. In addition, 13% of work-related accidents result from the inappropriate use of PPE [3].

The use of personal protective equipment has been identified as an important hazard control strategy in work environments where it may not be practical to adopt other strategies, and there is a great concern however that PPE usage remains low [4]. Evidence indicated that workers use of PPE is influenced by various factors. It has been reported that the influencing factors include sociodemographic factors, perception about occupational disease, and expectations of the benefits and barriers of PPE use [5, 6].

In Ethiopia, reports indicated that only 5 to 10% of workers have access to occupational health services in their respective workplaces [7]. But nearly half (44.66%) of the workers in Ethiopia have experienced occupational injury. Upon reviewing several literature studies about occupational health and safety among factory workers for this study, the PPE utilization rate typically ranges between 20.6% and 82.4% [7–10].

## 2. Methods

An institution-based cross-sectional quantitative study was employed from April 1 to May 1, 2021, in Debre Birhan North Shoa, Amhara region, Ethiopia. Based on CSA estimation, the town has a total population of 114,652. Among these, 51,843 are men and 62,809 are women. It is located at 130 km northeast of the capital Addis Ababa and has a total of 22 large scale factories. These factories are mainly involved in the production of processed food, textile, beverage, glass, and other products. The town is one of the most preferred investment destinations in the country. Thus, the study was conducted at selected large-scale factories in Debre Birhan town.

### 2.1. Source Population

All large-scale factory workers in Debre Birhan town were considered as a source population.

### 2.2. Study Population

Factory workers working in the selected factories in Debre Birhan town who could meet the inclusion criteria.

### 2.3. Study Unit

Selected factory workers working in the selected factories in Debre Birhan town who could meet the inclusion criteria.

### 2.4. Inclusion Criteria

All factory workers that were present at work during the study period were considered for the study.

### 2.5. Exclusion Criteria

Factory workers working in quality control and finishing section in beer and blanket factories were, respectively, excluded.

### 2.6. Sample Size Determination

The sample size for the dependent variable was calculated using single population proportion formula based on the following assumptions. Utilization of PPE was taken from a study done at Kombolcha textile factory which was 58.2% [11], level of confidence of 95%, margin of error of 5%, and nonresponse rate of 10% and gives a sample size of 412. Similarly, for associated factors by taking significantly associated variables with multivariate analysis in different studies, the sample size was calculated by using Epi Info 7 STATCAL software, cohort or cross-sectional study.

### 2.7. Sampling Technique and Procedure

A total of 22 large-scale factories in Debre-Birhan town were considered for sampling. Based on the assumption of 30% representatives, 7 factories were selected by simple random selection method. Before the data collection was started, all the necessary PPE for the workers working in different sections of the factory were identified based on literatures [12, 13]. Workers utilizing the similar type of PPE were considered for this study. The required sample size was taken proportional to the size of the selected factories. To get the individual sample at the selected factory, systematic random sampling was conducted by using the total number of factory workers who have worked during the study period and number of sample required in each selected factory. After getting the sampling fraction in the selected factory the first participant was obtained by lottery method among the first “k” units.

### 2.8. Data Collection Method

Interviewer-administered structured questionnaire was used to collect the required quantitative information on utilization and determinates of PPE use. Questionnaire was developed after review of similar literatures [11, 14, 15]. It includes socio-demographic characteristics of the workers, knowledge about PPE and occupational exposures, and seven HBM construct measures, namely, perceived susceptibility of occupational-related health problems, perceived severity of occupational-related health problems, perceived benefits of using PPE, perceived barriers to using PPE, perceived self-efficacy of using PPE, and cues to action of using PPE. The constructs of HBM were answered on a 5-point Likert's scale (1 = strongly disagree; 5 = strongly agree). The score of each participant was summed to get a score for each construct.

The questionnaire is prepared in English and translated to Amharic. The required data were collected by Four Environmental health professionals who are worked in woreda health office and health center. Training was provided for data collectors on different issues regarding the research.

### 2.9. Data Processing and Analysis

The collected data were checked for its completeness manually; data entry was done using EpiData version 3.1. The entered data was exported to SPSS version 21 for further cleaning and analysis. Data editing, coding, checking, and organization were done to transform the data into suitable format for further analysis. The model fitness was checked by Hosmer Lomeshow goodness of fit test. Assumptions of model including Multicollinearity and outlier were checked by variance inflation factor (VIF) and normal P-P plot, respectively. All independent variables were fitted separately into binary logistic regression model to evaluate the degree of association with utilization of PPE. The variables with a *p* value<0.20 was fitted to multiple logistic regression model. Then, AOR value with 95% CI was calculated to identify independent variables which were significantly associated with PPE utilization, a *p* value <0.05 was used as level of significance for the final qualifiers as factors associated with PPE utilization.

## 3. Result

### 3.1. Sociodemographic Characteristics of Factory Workers

A total 412 factory workers were involved in this study with 100% response rate. As shown in Table 1, the participants were predominantly male (66.3%). The age of the participants ranged from 18 to 57 with the mean age of 29.4 (±7.3) years. A majority of (97.3%) the participants were employed on a permanent basis with a small percentage of temporary workers 11 (2.7%). Concerning marital status of the workers, 47.3% were married (Table 1).

### 3.2. PPE Utilization Status

Overall, 41.7% (95% CI, 37.1–46.1) of the factory workers had good PPE utilization. The utilization was highly varied among the type of PPE. Respirator was found to be the most utilized PPE with a mean utilization score of 3.94 ± 1.42 followed by coverall. On the other hand, ear protector (1.14 ± 0.62) was found to be the least utilized PPE. Helmet, eye protector, coverall, safety shoe, and glove had a mean utilization score of 2.11 ± 1.26, 2.53 ± 1.63, 3.42 ± 1.71; 2.95 ± 1.82, and 2.41 ± 1.64, respectively (Table 2).

### 3.3. Knowledge towards PPE Utilization

In this study, 367 (89%) of workers knew that PPE can prevent work related injury and illness, of them, 164 (39.8%) were good PPE utilizers. Generally, in this research, 342 (83%) of factory workers had good knowledge about PPE, while the remaining 70 (17%) workers had poor knowledge regarding PPE utilization.

### 3.4. Perception towards Occupational Disease and PPE Utilization

Concerning perception of workers towards PPE utilization and occupational disease, it was measured using HBM constructs and treated as a continuous variable as shown in Tables 3 and 4. The mean score of perceived susceptibility was found to be 17.1(±8.7). For perceived severity, possible values range from 7 to 35 and the mean score was 20.2(±11.4). In addition, the mean scores of perceived benefit, perceived barrier, cues to action, and perceived self-efficacy were found to be 10.5 (±5.8), 22.0 (±9.3), 29.2 (±8.9), and 15.7 (±7.6), respectively.

### 3.5. Multivariate Analysis of Associated Factors

Of the total eight potential candidate predictors of PPE utilization of factory workers that were included in the multiple logistic regression model, only four (i.e., perceived susceptibility, perceived severity, self-efficacy, and perceived barriers) were found as predictors of PPE utilization (Table 5).

Generally, after controlling possible confounding factors, the result of the research indicated that per a unit increases in the total score of perceived susceptibility towards PPE utilization, the odds of using PPE increased by 20% (AOR = 1.2, 95%, CI (1.07–1.38)). Similarly, per a unit increases in the total score of perceived severity towards PPE utilization the odds of using PPE increased by 10% (AOR = 1.1, 95%, CI (1.01–1.16)). The other variable which independently associated with PPE utilization was perceived self-efficacy in which, per a unit increases in the total score of perceived self-efficacy towards PPE utilization the odds of using PPE was increased by 20% (AOR = 1.2, 95%, CI (1.08–1.34)). Perceived barrier was also found to be a negative predictors of PPE utilization in which per a unit increases in the total score of perceived barrier towards PPE utilization the odds of using PPE was decreased by 13% (AOR = 0.87, 95%, CI (0.80–0.95)).

## 4. Discussion

The finding of this research indicated that, only 172 (41.7%) of the workers were considered to have good PPE utilization. This finding was much lower than the study done in Thailand and Addis Ababa which indicated that 70.1% and 64.80% of the workers had good PPE utilization, respectively [16, 17]. On the contrary, the finding of this research was higher than the study done in Gujarat, India, that showed only 25% of the workers were considered to have good PPE utilization [18]. This difference might be due to methodological differences, like study population and methods of data collection, and workplace conditions, employees' level of awareness on hazard control and disease prevention.

The use of PPE varied considerably depending on the item examined with the respirator and overall being the most commonly used protective items. The finding was inconsistent with the study done in Hawasa town among Wood and Metal Worker that showed eye protector and safety shoe were the most utilized protective items [19]. On the other hand, in this research ear protector was found to be the least utilized type of PPE. The finding was different with the study done in Missouri, USA, that indicates helmet was found to be the least utilized PPE type [20]. This difference might be due to the difference in the nature of factories considered in the study.

In this research, none of the sociodemographic factors were significant at a *p* value of <0.05. This finding was inconsistence with the study done in different countries. For example, a research done in Uganda showed that, among the sociodemographic factors sex was found to be a significant predictors of PPE utilization in which female respondents were used PPE more than male respondent (AOR) = 6.64; 95% CI: 1.55–28.46 [21]). Similarly in Nepal female respondents were used PPE 3.65 times than male (AOR) = 6.64; *p*=0.031) [22]. This difference might be due to the difference in educational level and culture of the participants.

Another sociodemographic factor which was found to be insignificant predictors of PPE utilization was age of the workers. The finding was consistent with the study done in Uganda and Mombasa County, Kenya [21, 23]. But finding of this research regarding age was against with different research. For instance a research conducted in Indonesia among a sample of 200 workers indicated that workers who have the age of greater than 30 years have the possibility to use PPE 7.54 units higher than those below 30 years [24].

Similarly, employment form, income, working experience, and marital status was among the sociodemographic factors that were not predictors of PPE utilization. Similar finding was obtained from a study conducted in Kenya and Addis Ababa, in which employment form, income, working experience, and marital status were not determinant factors of PPE utilization [23, 25].

Regarding the HBM constructs, perceived susceptibility, perceived severity, perceived self-efficacy, and perceived barriers were significantly associated with PPE utilization. On the other hand, perceived benefit and cues to action towards PPE utilization were not found to be independent predictors.

Perceived susceptibility of occupational illness and injury has shown statistically significant association with PPE utilization. The study showed that, as a unit increase in total score of perceived susceptibility, the odds of utilizing PPE also increased by 20%. This finding is in line with different studies conducted in USA and Thailand [15, 17]. This might be explained as the study participants those having high susceptibility may belief that using PPE has the potential to protect work-related disease and injuries. Similarly, as a unit increases in total score of perceived severity, the odds of using PPE was increased by 10%. This finding is consistent with the studies conducted in Indonesia and USA. This might be due to the workers beliefs about the seriousness of the occupational illness, injury, and possible outcome of the disease. The other explanation may be high perceived susceptibility and severity towards occupational illness and injury may also increase the perceived threat of respondents; thus the participants could use PPE. In general, workers who perceived as they are highly susceptible to work related illness and injury and that they perceived work related disease is a serious disease, they would be more likely to utilize PPE.

The other predictor variable towards PPE utilization was perceived barrier, it was significantly associated with PPE utilization, and it indicated that, as a unit increases in sum score of perceived barriers, the odds of using PPE was decreased by 13%. Similar finding was reported from cross-sectional study conducted in Hawassa and Nigeria that showed barriers like inconvenience, unavailability, and increased cost were found to be predictors of PPE utilization [8, 26].

Self-confidence in using PPE (perceived self-efficacy) was found to be a significant predictor of PPE utilization, in which, per a unit increases in the total score of perceived self-efficacy towards PPE utilization the odds of using PPE was increased by 20% (AOR = 1.2, 95%, CI (1.082–1.349)). The possible justification might be People with high self-efficacy show elevated confidence in their skills and have no doubt about themselves. In these cases, factory workers consider the problems as a challenge, not a threat, and they actively search for new situations. In addition, high self-efficacy reduces fear of failure, increases the level of motivation, and improves problem-solving and analytical thinking abilities. In the same way, high self-efficacy in working a hazardous environment may promote the use of PPE.

On the other hand, perceived benefit (AOR = 1.05, 95%, CI (0.95–1.17, *p*=0.302)) were not found to be predictors of PPE utilization. This finding was in line with the study done in USA, in which perceived benefit towards using PPE was not found to be independent predictors. Similarly, cues to action were not predictors of PPE utilization. The finding is inconsistent with the study done in Indonesia to identify factors influencing the use of PPE ((OR = 7.17; 95%CI = 2.17 to 23.62; *p*=0.001). This inconsistency may be due to the difference of educational level, media exposure, and culture of the participants.

## 5. Conclusion

The finding revealed that there was good PPE utilization among large-scale workers. Perceived susceptibility, perceived severity, perceived self-efficacy, and perceived barrier were found to be strong predictors of PPE utilization.

### 5.1. Recommendations

- For health professionals delivering health education or health information should create an awareness to increase personal protective equipment utilization
- They should also provide special emphasis to susceptibility and severity of occupational disease and injuries
- For regulatory bodies, workers association and other stake holders have to work hard towards increasing personal protective equipment utilization focusing on modification of barriers and increasing self-confidence of workers in using PPE
- For factory managers in order to increase personal protective equipment utilization, focus should be on reducing barriers of PPE utilization

### 5.2. Limitation of the Study

Since the study is cross-sectional, it does not show cause and effect relationship between dependent and independent variables.

## Figures and Tables

**Table 1:** Sociodemographic characteristics of large scale factory workers in Debre-Birhan town, Ethiopia, June 2021.

Variable	Categories	Frequency	Percent
Sex	Male	273	66.3%
	Female	139	33.7%
Age	18–28	220	53.4
	29–39	140	34.0
	>39	52	12.6
Educational level	Primary	10	2.4
	Secondary	69	16.7
	Diploma	203	49.3
	First degree and above	130	31.6
Marital status	Married	195	47.3
	Single	213	51.7
	Others^a^	4	0.9
Employment form	Temporary/contract	11	2.7
	Permanent	401	97.3
Working experience	<5	321	77.9
	5–10	60	14.6
	>10	31	7.5
Income	500–2500	151	36.7
	2501–4500	118	28.6
	4501–6500	127	30.8
	>6501	16	3.9

^a^Widowed and divorced.

**Table 2:** PPE utilization frequency score of large-scale factory workers in Debre-Birhan town, Ethiopia, June 2021.

Type of PPE	Frequency (%)					Mean	SD
	Never	Rarely	Sometimes	Often	Always		
Helmet	178 (43.2)	105 (25.5)	69 (16.7)	25 (6.1)	35 (8.5)	2.11	1.26
Eye protector	160 (38.8)	103 (25)	30 (7.3)	10 (2.4)	109 (26.5)	2.53	1.63
Ear protectors	385 (93.4)	14 (3.4)	2 (0.5)	4 (1)	7 (1.7)	1.14	0.62
Respirator	53 (12.9)	22 (5.3)	46 (11.2)	67 (16.3)	224 (54.4)	3.94	1.42
Coverall	85 (20.6)	86 (20.9)	21 (5.1)	10 (2.4)	210 (51)	3.42	1.71
Safety shoe	168 (40.8)	32 (7.8)	27 (6.6)	24 (5.8)	161 (39.1)	2.95	1,82
Glove	215 (52.2)	17 (4.1)	60 (14.6)	35 (8.5)	85 (20.6)	2.41	1.64

**Table 3:** Health belief model construct response of factory worker in Debre-Birhan town, Ethiopia, June 2021.

HBM constructs	Strongly disagree	Disagree	Neutral	Agree	Strongly agree
	*F* (%)	*F* (%)	*F* (%)	*F* (%)	*F* (%)
*Perceived susceptibility*					
It is extremely likely I will get occupational illness or injury in the future	122 (29.6)	87 (21.1)	51 (12.4)	40 (9.1)	112 (27.2)
I think my chance of developing occupational illness is grate	83 (20.1)	122 (29.6)	60 (14.6)	35 (8.5)	112 (27.2)
There is a good possibility I will get occupational illness in the next few years	77 (18.7)	98 (23.8)	40 (9.7)	115 (27.9)	82 (19.9)
I know predecessors in this career field who got an occupational illness	115 (27.9)	92 (22.3)	47 (11.4)	23 (5.6)	135 (32.8)
I think small exposures to occupational chemicals or noise will lead me to an illness	139 (33.8)	81 (19.7)	4 (1.0)	84 (20.4)	104 (25.2)
I am more likely than the average worker to have occupational illness or injury	152 (36.9)	115 (27.9)	16 (3.9)	21 (5.1)	108 (26.2)

*Perceived severity*					
The thought of getting an occupational illness is deeply concerns me	163 (39.6)	92 (22.3)	16 (3.9)	15 (3.6)	126 (30.6)
If I developed an occupational illness, my career would be in danger	157 (38.1)	50 (12.1)	13 (3.2)	16 (3.9)	176 (42.7)
Problems I would experience from an occupational illness would last a life time	73 (17.7)	139 (33.7)	9 (2.2)	49 (11.9)	142 (34.5)
An occupational illness will lead to permanent changes in my health	204 (49.5)	54 (13.1)	5 (1.2)	19 (4.6)	130 (31.6)
My financial security would be endangered if I developed an occupational illness	146 (35.4)	56 (13.6)	19 (4.6)	13 (3.2)	178 (43.2)
I believe I could die prematurely if I developed an occupational illness	131 (31.8)	89 (21.6)	7 (1.7)	33 (8.0)	152 (36.9)
I am afraid to even think about getting an occupational illness	85 (20.6)	131 (31.8)	29 (7.0)	2 (0.5)	165 (40)

*Perceived benefits*					
Wearing PPE will prevent me from occupational illness	188 (45.6)	61 (14.8)	16 (3.9)	66 (16)	81 (19.7)
PPE prevents exposure to the kinds of hazards you are around on the job	112 (27.2)	116 (28.2)	43 (10.4)	60 (14.6)	81 (19.7)
I worry about getting an occupational illness when I do not wearing PPE	144 (35)	120 (29.1)	15 (3.6)	33 (8.0)	100 (24.3)
I benefit by wearing PPE	118 (28.6)	131 (31.8)	12 (2.9)	30 (7.3)	121 (29.4)

*Perceived barriers*					
When I wear PPE I feel uncomfortable	136 (33.0)	21 (5.1)	11 (2.7)	142 (34.5)	102 (24.8)
When I wear PPE, it interferes with my ability to do my job	124 (30.1)	74 (18.0)	12 (2.9)	107 (26.0)	95 (23.1)
PPE is not always available to me	115 (27.9)	66 (16.0)	11 (2.7)	138 (33.5)	82 (19.9)
When I wear PPE co workers would make fun of me	85 (20.6)	107 (26.0)	7 (1.7)	74 (18.0)	139 (33.7)
My supervisor is aware of my compliance with PPE guidelines	66 (16.0)	70 (17.0)	14 (3.4)	118 (28.6)	144 (35.0)
I would need to develop a new habit for wearing PPE, and that is difficult to me	80 (19.4)	113 (27.4)	16 (3.9)	80 (19.4)	123 (29.9)
Wearing PPE is just too inconvenient for you	48 (11.7)	158 (38.3)	12 (2.9)	58 (14.1)	136 (33.0)

*Cues to action*					
A reminder from my supervisor everyday would be important to wear of PPE	135 (32.8)	30 (7.3)	97 (23.5)	54 (13.1)	96 (23.3)
My supervisor checking on me would improve you to wear of PPE	33 (8.0)	109 (26.5)	73 (17.7)	89 (21.6)	108 (26.2)
My employer is important for wearing PPE	25 (6.1)	93 (22.6)	90 (21.8)	95 (23.1)	109 (26.5)
Posters in my factory would serve as important reminders to wear PPE	46 (11.2)	92 (22.3)	46 (11.2)	105 (25.5)	123 (29.9)
The threat of disciplinary action is an important factor in ensuring I wear PPE	66 (16.0)	72 (17.5)	86 (20.9)	96 (23.3)	92 (22.3)
Having PPE at location of hazard is critical to ensure that I wear it	72 (17.5)	62 (15.0)	54 (13.1)	123 (29.9)	101 (24.5)
If you see others wearing PPE in your area, then it reminds you to use it	110 (26.7)	24 (5.8)	62 (15.0)	65 (15.8)	151 (36.7)
Regular and frequent education on the importance of PPE improves how often I wear it	86 (20.9)	70 (17.0)	38 (9.2)	46 (11.2)	172 (41.7)
My supervisor sets the example on wearing PPE when being exposed to hazard	77 (18.7)	113 (27.4)	40 (9.7)	32 (7.8)	150 (36.4)

*Self-efficacy*					
I am confident that I remember to use PPE when I am exposed to hazards at work	108 (26.2)	89 (21.6)	11 (2.7)	38 (9.2)	166 (40.3)
I am confident I can obtain the proper PPE when I am exposed to hazards at work	101 (24.5)	83 (20.1)	40 (9.7)	25 (6.1)	163 (39.6)
I am confident that my job performance will NOT be impacted by wearing PPE	104 (25.2)	76 (18.4)	36 (8.7)	19 (4.6)	177 (43.0)
I am confident that the PPE I use when I am exposed to hazard is the proper equipment to protect my health	25 (6.1)	163 (39.6)	19 (4.6)	24 (5.8)	181 (43.9)
I am confident that after wearing the proper PPE throughout my career will prevent me from getting an occupational illness	156 (37.9)	17 (4.1)	7 (1.7)	70 (17.0)	162 (39.3)

**Table 4:** Health belief model construct score of large-scale factory worker in Debre-Birhan town, Ethiopia, June 2021.

	Perceived susceptibility	Perceived severity	Perceived benefit	Perceived barrier	Cues to action	Perceived self- efficacy
Mean	17.1	20.2	10.5	22.0	29.2	15.7
Min	6.00	7.00	4.00	7.00	9.00	5.00
Max	30.00	35.00	20.00	35.00	45.00	25.00
SD	8.7	11.4	5.8	9.3	8.9	7.6

**Table 5:** Multivariate analysis of factors associated with PPE utilization among factory workers in Debre-Birhan town, Ethiopia, June 2021.

Variable	Categories	COR	95% CI		*p* value	AOR	95% CI		*p* value
			*L*	*U*			*L*	*U*	
Sex	Male	1.43	0.944	2.19	0.09	0.77	0.26	2.28	0.645
	Female	1.00				1.0			
Income	500–2500	0.540	0.179	1.63	0.274	0.05	0.00	56.55	0.421
	2501–4500	0.233	0.076	0.717	0.011	0.06	0.00	69.48	0.455
	4501–6500	0.201	0.066	0.619	0.005	0.06	0.00	73.64	0.454
	>6501	1.00				1.00			
Knowledge	Good	2.58	1.43	4.64	0.002	1.9	0.51	7.34	0.332
	Poor	1.00				1.00			
Perceived susceptibility		1.465	1.364	1.573	<0.001	1.2	1.07	1.38	0.002^*∗*^
Perceived severity		1.311	1.248	1.377	<0.001	1.1	1.01	1.16	0.012^*∗*^
Perceived benefit		1.059	1.024	1.095	0.001	1.05	0.95	1.17	0.302
Perceived barrier		0.713	0.670	0.758	<0.001	0.87	0.80	0.95	0.003^*∗*^
Self-efficacy		1.422	1.338	1.510	<0.001	1.2	1.08	1.34	0.001^*∗*^

^*∗*^Significant at *p* < 0.05.

## Data Availability

The data collected for this study can be obtained from the first or last author upon a reasonable request.

## Abbreviations

PPE: Personal protective equipment
HBM: Health belief model
OSHA: Occupational Health and Safety Act.
